# Recovery from bleaching is mediated by threshold densities of background thermo-tolerant symbiont types in a reef-building coral

**DOI:** 10.1098/rsos.160322

**Published:** 2016-06-29

**Authors:** Line K. Bay, Jason Doyle, Murray Logan, Ray Berkelmans

**Affiliations:** 1 Adaptation and Resilience of Coral Reefs to Climate Change, Australian Institute of Marine Science, PMB 3 Townsville MC, Townsville, Queensland 4810, Australia; 2 ARC Centre of Excellence for Coral Reef Studies, James Cook University, Townsville, Queensland 4811, Australia

**Keywords:** *Symbiodinium*, coral, shuffling, thermal stress, physiological adaptation

## Abstract

Sensitive molecular analyses show that most corals host a complement of *Symbiodinium* genotypes that includes thermo-tolerant types in low abundance. While tolerant symbiont types are hypothesized to facilitate tolerance to temperature and recovery from bleaching, empirical data on their distribution and relative abundance in corals under ambient and stress conditions are still rare. We quantified visual bleaching and mortality of coral hosts, along with relative abundance of C- and D-type *Symbiodinium* cells in 82 *Acropora millepora* colonies from three locations on the Great Barrier Reef transplanted to a central inshore site over a 13 month period. Our analyses reveal dynamic change in symbiont associations within colonies and among populations over time. Coral bleaching and declines in C- but not D-type symbionts were observed in transplanted corals. Survival and recovery of 25% of corals from one population was associated with either initial D-dominance or an increase in D-type symbionts that could be predicted by a minimum pre-stress D : C ratio of 0.003. One-third of corals from this population became D dominated at the bleached stage despite no initial detection of this symbiont type, but failed to recover and died in mid to late summer. These results provide a predictive threshold minimum density of background D-type symbionts in *A. millepora,* above which survival following extreme thermal stress is increased.

## 1. Introduction

Coral cover is declining on reefs around the world through the effects of global and local stressors (mostly) associated with human activities [1–3]. A major source of variation in coral physiology and stress tolerance results from their symbiosis with photosynthetic microalgae (*Symbiodinium* spp.). Coral-associated *Symbiodinium* are genetically diverse [4] and clades, types and ecomorphs (within types) have pronounced effects on their hosts (e.g. [5,6]). Differences in function and performance by *Symbiodinium* [7] can affect the growth [8], disease susceptibility [9,10] and thermal tolerance [5,6,11] of coral hosts. Significant variation exists in associations between coral host and *Symbiodinium* types [12,13] and may correlate with environmental variation suggesting the potential for local adaptation in coral–symbiont associations. As our understanding of the distribution of *Symbiodinium* within coral species and among geographical locations increases, questions regarding their relative abundance during ambient and stressful conditions, and the effects on coral hosts' physiology and bleaching susceptibility can now be addressed [3].

The molecular tools used to quantify metazoan–microbial association (including coral–*Symbiodinium* associations) have undergone a revolution in recent years with quantitative polymerase chain reaction (qPCR) and next-generation sequencing vastly increasing our ability to identify and quantify *in hospite* microbial communities [14,15]. Deep sequencing of DNA-barcoded bacteria has revealed the presence of abundant and rare bacterial taxa in tropical sponges that are vital for the health of their hosts [16]. Temporal variation in the relative abundance of microbial taxa, including pathogenic strains, has been associated with environmental stress [17] and can be detected before visual signs of coral stress emerge. Likewise, the detection of low-abundance microbes and *Symbiodinium* in corals has vastly improved with the much higher detection rate and quantitative accuracy offered by qPCR relative to gel fingerprinting [18].

Predicting the future of reef corals under ongoing climate change depends to a large extent on how accurately processes of genetic, epigenetic and physiological adaptation can be modelled (e.g. [19]). Considered particularly important is ‘shuffling’, a process whereby the relative abundance of sensitive and more tolerant symbiont types changes following acute stress (e.g. [20,21]). Shuffling can increase stress thresholds by 1–1.5°C on time scales that are relevant to the rates of environmental change currently being experienced [5,11,19,22,23]. While a change from sensitive symbiont ‘C’ to tolerant ‘D’ has been observed in the field following bleaching [23,24], it is not a ubiquitous response [25–27] and this makes projection modelling a challenging exercise [19]. Therefore, defining and measuring predictors of shuffling and survival from bleaching stress in wild populations of corals is a research priority.

We examine here the absolute and relative changes in C and D symbionts in *Acopora millepora* corals from three populations when exposed to warm summer temperatures that exceeded their upper long-term temperature regimes by 2–4°C for an extended period. This coral species is common on the Great Barrier Reef (GBR) where it is dominated by *Symbiodinium* ITS-1 types: C1, C2, C2* and D (ITS-2 equivalents: C1, C3, C131 and D). Background types have been documented in colonies from many locations, particularly turbid inshore reefs, but their roles in bleaching susceptibility, shuffling potential and survival are not yet clear [28]. We test the role of low-abundance symbiont types in bleaching intensity, survival and recovery of *Acopora millepora.* We show that the relative abundances of symbiont types are dynamic and that coral survival and recovery following bleaching is associated with an increase in the relative abundance of D-type symbionts through shuffling. We define a threshold minimum density of background D above which recovery and survival was enhanced and discuss ways in which this new knowledge can be applied in natural resource management.

## 2. Material and methods

### 2.1. Coral transplantation

Temporal changes in C and D *Symbiodinium* cell abundance were examined in adult colonies (20–30 cm in diameter) of *Acropora millepora* at six sampling times that encompassed a summer season. Thirty-two colonies were collected from a depth of 3–7 m at North Keppel Island in the southern inshore GBR (23.2° S; 151.0° E) on 29 March 2005, 33 colonies from Davies Reef in the central GBR (18.8° S; 147.6° E) and 17 from Magnetic Island were collected on 2 April 2005. All colonies were placed on wire mesh racks at 4 m depth in Geoffrey Bay, Magnetic Island (19.2° S; 146.8° E) following the methods in [5]. Samples (single branches) were collected and preserved in 100% EtOH for symbiont genotyping at the time of transplanting (autumn 2005: 2 April 2005) and a further five times over the following 13 months (spring: 14 September 2005; early summer: 21 December 2005; mid-summer: 16 January 2006; late summer: 14 February 2006; and autumn 2006: 24 April 2006). Partial and whole-colony mortality and visual colour score (1–5 scale on the coral colour chart [29]) were also estimated for all colonies at the sampling times. Three hundred and seventy-seven samples were collected, but 32 samples were accidentally lost and hence not included in molecular analyses.

**Figure 1.**
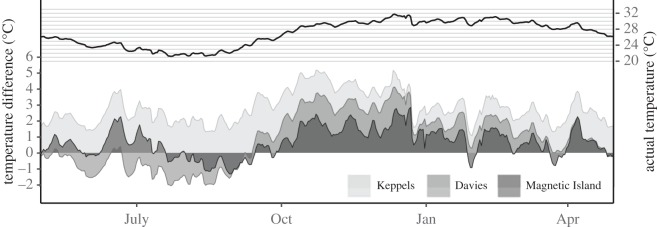
Temperature profile at Magnetic Island during the experiment (top) and population-specific deviation of average annual temperature profile from actual temperature experienced during the experiment.

Long-term temperature regimes for these sites are available from *in situ* temperature loggers (http://data.aims.gov.au/aimsrtds/datatool.xhtml). A 15-year daily mean temperature climatology was computed for each site using 48–144 measurements per day to indicate the normal thermal range for each population, the daily mean temperatures for the 13 month transplant period at Magnetic Island from April 2005 to April 2006 and the departure of ambient temperatures from historical averages (figure 1). Bleaching and mortality thresholds *sensu* Berkelmans [30,31] were used to determine the date at which ambient temperatures at the transplant location exceeded population-specific stress thresholds. Briefly, these thresholds were developed with time--temperature curves and observed bleaching and mortality data for 13 reefs following the 1998 mass-bleaching event. They have since been validated with data from subsequent bleaching events on the GBR and can more accurately predict bleaching and mortality at local scales compared with other bleaching metrics [31].

### 2.2. Symbiont genotyping and density

DNA was extracted from 345 crushed coral samples with a MoBio plant kit following the manufacturer's protocols.

Symbiont genotyping was achieved on 330 samples with DGGE of the ITS-2 region [32] using a denaturing gradient of 30–50% (15 samples did not amplify). Direct sequencing of excised DGGE bands distinguished C1, C3 and C131 but not D, D1 or D1a types [33]. Only a single-clade C type was evident from DGGE analysis in all samples (see the electronic supplementary material for further details).

qPCR of the *Actin* locus [34] was used to quantify coral host, *Symbiodinium* clade C and *Symbiodinium* clade D on 332 samples following Mieog *et al*. [35] (details in the electronic supplementary material, File 1; 13 samples did not amplify). Actin copy number was determined from one coral sample each with ITS-2 symbiont types C1, C3 or C131 (accession numbers KJ612067–KJ612069; details in the electronic supplementary material, File S1). The results were: clade C3 = 1.0 ± 0.5, C1 = 5.1 ± 1.2 and C131 = 4.3 ± 1.0 (mean ± s.d.). The copy number for C1 was within the margin of error of that determined by Mieog *et al*. [35] for the same symbiont type, coral host species and sampling location (7.0 ± 2.9). For clade D, we used a copy number of 1.0 ± 0.7 and one for host *Actin* as determined by Mieog *et al*. [35].

### 2.3. Symbiont : host and symbiont D : C ratios

Symbiont types C or D to host ratios were calculated with the formula $S_{\left[C\;\mathrm{or}\;D\right]}:H\;\mathrm{ratio}=[2^{C_{T}(H)-C_{T}(S_{\left[C\;\mathrm{or}\;D\right]})}/\mathrm{actin}\;\mathrm{copies}\;\mathrm{per}\;\mathrm{cell}]\times 2$ with further details and copy number correction provided in the electronic supplementary material S1. The overall symbiont : host (S : H) ratio was obtained from the sum of clade C and D ratios (S[_C_] : H + S[_D_] : H). Ratios of *Symbiodinium* clade D : C were calculated with the formula $D:C\;\mathrm{ratio}=[2^{C_{T}(\mathrm{clade}\;C)-C_{T}(\mathrm{clade}\;D)}\times \mathrm{CNR}].$ CNR is the copy number ratio of clade C to clade D, and this correction was applied on a sample by sample basis based on dominant C-type determined by DGGE and cross-referenced by direct sequencing.

### 2.4. Statistical analysis

All statistical analyses were performed in R v. 2.2.2 [36], and raw data and scripts are deposited in Dryad [37]. The D : C ratios were highly skewed and standard square root and logarithmic transformations failed to normalize the data. We therefore applied a custom transformation that consisted of 20 bins (electronic supplementary material, table S1) and, subsequently, the data met the linear model assumptions. This approach had the advantage that it provided adequate granularity of the data (particularly for low D : C estimates of interest here) and naturally constrained the data between 0 and 1 (i.e. all C or all D; more detail in the electronic supplementary material). No transformations were applied to the visual and mortality data as they were tested against suitable distributions (i.e. binomial and quasi-Poisson).

Trends in visual colour, coral mortality and symbiont densities (D- and C-specific S : H ratios) over time from each source population were modelled via generalized additive mixed effects models (GAMM) using *mgcv* [38] and generalized linear mixed effects models (GLMM) [39,40] using MASS [41] incorporating samples as random effects and first-order autoregressive correlation structure to account for the dependency structure and temporal autocorrelation. GAMMs included a thin-plate smoothing spline function of time and the degree of smoothing was established via general cross-validation. Time smoothers lacking inferential support for nonlinear trends (effective degrees of freedom not significantly different from 1) were thereafter modelled via GLMM.

To examine the role of initial symbiont density on bleaching level a linear model compared square root-transformed symbiont densities (i.e. S : H (C + D)) at the beginning of the experiment (April 2005) among the three populations and their bleaching level in December 2005 (also square root-transformed). Generalized change-point (piece-wise) [42] regression models were used to model December S : H ratio (i.e. bleaching level) against April and September D : C ratios (see the electronic supplementary material, S1). The value of the change-point was estimated by iterative optimization of the linear model to minimize model deviance and 95% CIs were generated by bootstrapping the optimization process (leaving one observation out each time).

## 3. Results

**Figure 2.**
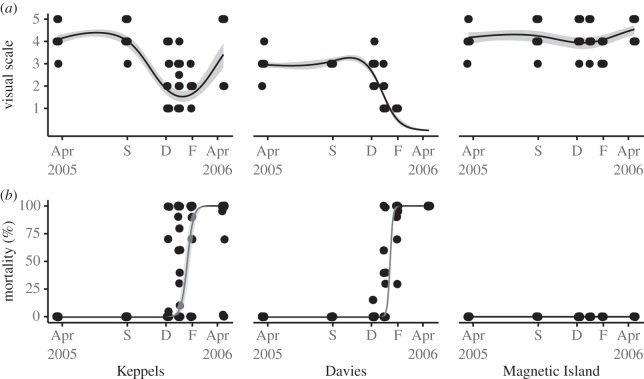
Actual and modelled (*a*) visual bleaching condition (health chart scale 1–5) and (*b*) mortality (%) show contrasting trends in the three populations over time. Keppel and Davies Reef corals bleached and showed high mortality during the summer months. Surviving Keppel Island corals recovered in late summer. No bleaching and very low mortality were observed in Magnetic Island corals. Points represent individual colonies and shaded areas are residual error variances.

Water temperatures at the transplant site were within 2°C of local long-term averages for corals from the three source populations from April to September 2005 but elevated by up to 4°C for extended periods between September and December 2005 (figure 1). In agreement with visual condition, population-specific temperature thresholds for bleaching were exceeded on 8 November and 14 November 2005 for Keppels and Davies Reef corals and mortality thresholds (*sensu* [31]) were exceeded on 6 December for Keppels, but 14 November for Davies Reef corals. Magnetic Island native corals did not exceed their bleaching thresholds during this study and were not observed to pale or bleach (figure 2*a*). Mortality in December, January and February varied among populations with 8, 40 and 75% in Keppels and 3, 11 and 96% in Davies Reef, respectively. By April 2006, all Davies Reef colonies were dead, whereas 25% of Keppels corals survived. No mortality was observed in Magnetic Island corals (figure 2*b*).

**Figure 3.**
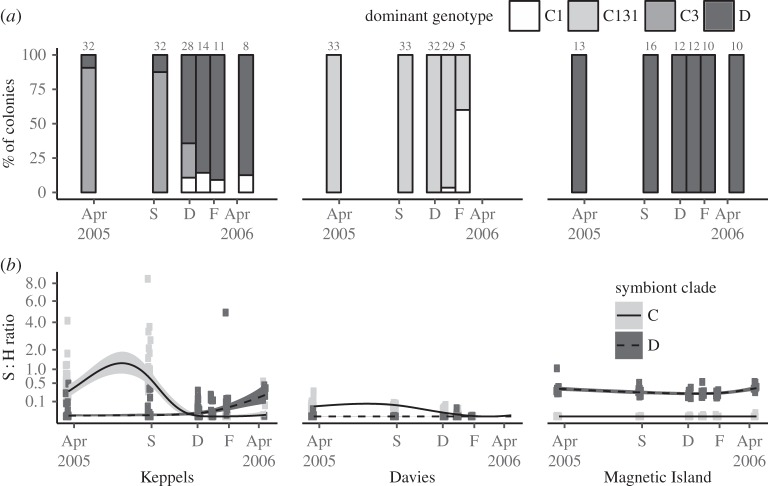
Symbiont associations in coral colonies from three populations across an annual cycle. (*a*) Dominant symbiont type as determined by DGGE of ITS-2 (sample size indicated above bars). (*b*) Relative abundance of C and D type symbionts in relation to host cell abundance determined by qPCR of host and symbiont Actin genes.

We detected three dominant ITS-2 DGGE symbiont types (C1, C131 and D) in the experimental corals and a single type was detected in 95% of samples (more details in the electronic supplementary material). Dominant ITS-2 DGGE symbiont type changed substantially in the Keppels corals over the course of the experiment, but not in Davies Reef and Magnetic Island corals (figure 3*a*). The Keppels population was mostly C3-dominant at the time of transplanting (91%) with the remaining D-dominant (figure 3*a*). A substantial shift in the dominant symbiont community took place between September and December 2005 with 64% becoming D-dominant, while 25% and 11% of colonies were C3- and C1-dominated, respectively (figure 3*a*). Three weeks later (January 2006) 12 of the surviving 14 colonies were D-dominant, the other two were C1-dominant and had very low symbiont densities (less than 0.006 symbionts per host cell). This proportion remained until the end of the experiment. All Davies Reef colonies were C131-dominant and remained so until the corals were white bleached in January 2006 when one colony became C1-dominant. Although most colonies died before the February 2006 sampling event, three of the five remaining colonies became C1-dominant, albeit at very low symbiont densities (S : H (C + D) < 0.0001). The Magnetic Island population remained D-dominant throughout the experiment.

S : H (C + D) ratios varied among colonies depending on their condition and the median S : H (C + D) of visually bleached corals (visual score 1–2) was 0.0003, whereas healthy corals (visual score 4–5) had a median S : H (C + D) of 0.30. Both C and D were detected in 138 samples, whereas only C or only D were observed in 87 and 107 samples, respectively. Observed and modelled S : H ratios revealed temporal dynamics among populations and symbiont types (figure 3*b*). Combined symbiont densities in April 2005 were higher in the Magnetic Island than the Keppels population (*b* = 0.186, *t*_62_ = 4.815, *p* < 0.05), yet not found to differ from those of the Davies Reef population (*b *= −0.019, *t*_62_ = −1.050, *p* > 0.05). C-types increased in the Keppel population over the winter period (April–September 2005) but declined in Keppels and Davies corals in early and mid-summer (December–January 2006) consistent with their population-specific predicted bleaching thresholds and the visual appearance of the corals (figure 2*a*). D-type symbionts increased in the Keppels population from the early summer (December 2005) and reached their highest densities in April 2006 but remained relatively constant at very low and very high levels in corals from Davies Reef and Magnetic Island, respectively (figure 3*b*). For the Keppels population, S : H ratios were at their lowest in December 2005 with a median combined C and D symbiont density of 0.003 symbionts per host cell but increased to a median S : H = 0.34 in the surviving colonies by April 2006. The Magnetic Island S : H ratios, however, remained high throughout the summer period consistent with their healthy visual appearance (median S : H (C + D) ratio in December = 0.21 and 0.38 in April 2006). By contrast, Davies Reef corals had a lower symbiont density in April 2005 (median S : H (C + D) = 0.04), likely due to naturally clearer waters at this location. This halved in early summer when corals started to pale and continued to decline until late summer (median S : H = 0.001) after which all colonies died (figure 3*b*). Pre-summer symbiont density (i.e. S : H (C + D) in April 2005) did not predict bleaching level in any of the three source populations (i.e. the partial slopes for the relationship between S : H in December and S : H in April 2005 and population interactions were Keppels: *b* = −0.08, *t*_62_ = −1.437, *p* > 0.05; Magnetic Island: *b* = 0.139, *t*_62_ = 0.561, *p* > 0.05; Davies Reef: *b* = −0.011, *t*_62_ = −0.049, *p* > 0.05, electronic supplementary material, figure S1).

**Figure 4.**
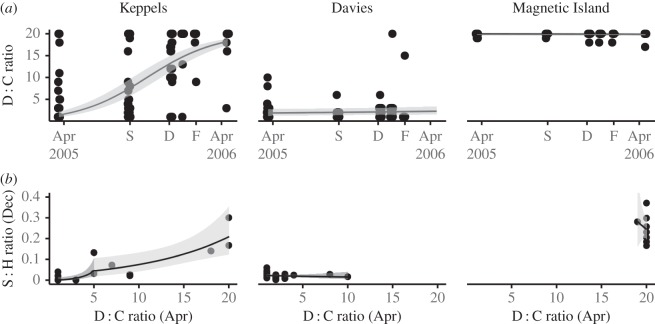
Symbiont community dynamics in coral colonies from three source populations. (*a*) Ratio of D to C symbionts across an annual cycle that included a very warm summer and coral bleaching. (*b*) Level of bleaching expressed as symbiont abundance in summer as a function of pre-bleaching D : C symbiont ratio.

**Figure 5.**
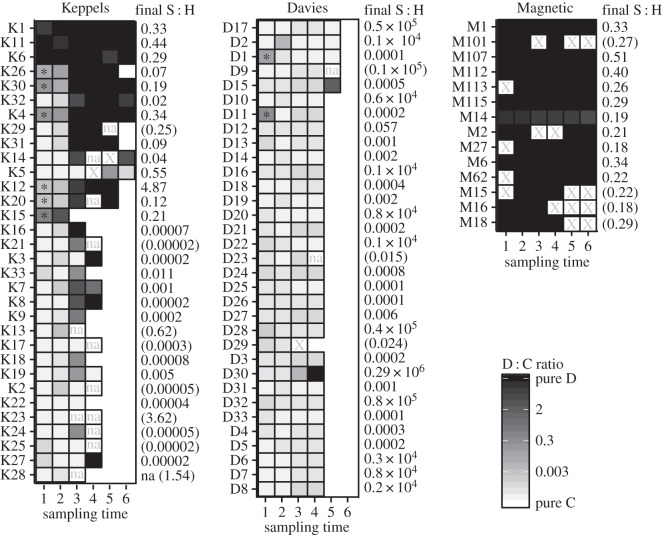
Colony level D : C ratio among sampling times and source populations including S : H (C + D) at the final sampling time (in parentheses if not from the last sample collected). Sampling times include in sequential order April, September, December 2005, January, February and April 2006. Missing data are indicated by X and non-amplifications with ‘na’. Asterisk indicates a D : C ratio above 0.003. No outline means that the colony was dead and no sample collected. D : C ratio was expressed as minimum D : C ratio of 20 bins (electronic supplementary material, table S1). Three samples from Magnetic Island contained 50% missing data and were not included.

Observed and modelled qPCR D : C ratios were consistent with the ITS-2 DGGE data with a substantial shift in the Keppels population from low D : C ratios at the start of the experiment to high D : C ratios at the end (figure 4*a*). The abundance of D increased in 24 colonies over the course of the experiment and all but one of the surviving colonies was D-dominated in April 2006 (three of these were D-dominated in April 2005). In the Keppels population, half of the eight surviving colonies bleached and recovered (i.e. S : H (C + D) < 0.003 in December 2005 and >0.15 in April 2006). These colonies generally started with higher background levels of D and shuffled from C to D dominance (figure 5). An additional 12 colonies increased their D abundance over the summer sampling times despite the absence of detectable levels of D at the start of the experiment. These colonies did not recover and died before the end of the experiment (i.e. final S : H (C + D) < 0.002; figure 5). Nineteen colonies from Davies Reef had detectable levels of D at the start of the experiment but only two increased their D : C ratios in January and February. These colonies were visually bleached, had low S : H (C + D) ratios (i.e. 0.0005 and 0.0011) and did not survive.

To identify the predictors of bleaching level, we examined the relationship between pre-experimental D : C ratios and mid-summer bleaching level (i.e. April 2005 D : C ratio versus December 2005 S : H ratio). For the Keppels population, we found a positive relationship between bleaching and the pre-experimental D : C ratios (figure 4*b*). Moreover, fitting a piece-wise (broken-stick) generalized linear model to the data indicated a break point (i.e. threshold) in D : C ratios where the behaviour of the relationship was different. Model deviance in linear optimization suggests that this threshold occurs at minimum D : C > 0.003 (figure 4*b*). For D : C ratios greater than this threshold, D : C ratio was associated with a gradual decline in S : H ratio (and thus bleaching). However, D : C ratios less than 0.003 were associated with a more dramatic decline in S : H ratio. A similar break point was evident with the September 2005 D : C ratios (electronic supplementary material, figure S2). We did not find a relationship between the D : C (April or September 2005) and S : H (December) for the Davies Reef population. No corals from Magnetic Island populations bleached and hence no relationship was found (figures 4*b* and 5; electronic supplementary material, figure S2).

## 4. Discussion

The application of sensitive molecular tools to understand the distribution and abundance of microbial symbionts in Metazoa has revealed their central role in species and ecosystem health [43]. The role of the dominant *Symbiodinium* type in coral host physiology and stress tolerance is well recognized [21]. However, many key questions regarding natural variability in symbiont distribution and abundance (both environmental and *in hospite*) remain, including the costs and benefits of symbiosis on coral performance and stress susceptibility under current and future environmental scenarios [3]. We add new insights to this emerging literature with a field experiment that demonstrates the dynamic nature of coral–*Symbiodinium* associations. We identify indicators of susceptibility to and survival from bleaching that may be used to monitor the status and trend in coral health and resilience in the face of ongoing environmental change.

The abundance of symbionts within corals' tissues can be highly variable in space and time, and can influence coral physiology including bleaching sensitivity (reviewed in [3,21]). Cunning & Baker [44] found more intense bleaching (i.e. reduced S : H ratio) in *Pocillopora damicornis* with abundant C-type *Symbiodinium* communities compared to those with less abundant C- or D-type communities. The density of symbionts pre-summer (both April and September 2005) did not predict the level of bleaching detected here. Our analyses did, however, reveal contrasting and dynamic patterns in the abundance of C- and D-type symbionts among colonies from the three source populations in response to seasonal and extreme summer temperatures. An increase in C-type symbionts was observed in corals from the Keppels after five months of transplantation but not in the Davies Reef population where C-type abundance remained stable until early summer (December 2005; figure 3*b*). This result might be due to symbiont-specific responses to the novel environment since the transplanted inshore and offshore populations were dominated by C3 and C131, respectively. The specificity of our primers, however, did not allow us to differentiate quantitatively among C-types. The abundance of symbiont types can also be dependent on the ambient temperature environment [45] which was on average 2.2°C above historical averages over winter for the Keppels corals where C-types became more abundant compared with 0.92°C cooler than average for the Davies Reef source population where C-type abundance remained constant. Symbiont densities may be enhanced by environmental nutrient levels [46] and are predicted to be higher on inshore reefs, as a function of higher dissolved nutrients often experienced there. We recorded higher S : H ratios in the corals from the two inshore locations (Keppels and Magnetic Island) compared to the offshore location (Davies Reef). Spatial variation in symbiont density has been hypothesized to underpin larger spatial scale patterns in bleaching on the GBR in 1998 and 2002 [47]. Our results emphasize the importance of the genetic identity of coral host and/or symbiont type rather than cell abundance for bleaching susceptibility and recovery.

Our results confirmed that D-dominated corals are more tolerant to elevated temperatures [5,23,24,48] with all D-dominated colonies but one resisting bleaching regardless of their source population and historical thermal regime*.* Interestingly, 37.5% of Keppels corals increased their abundance of D over time despite the absence of measurable levels of this type at the beginning of the experiment. This pattern was also found in *Montastraea cavernosa* by Silverstein *et al*. [48] and LaJeunesse *et al*. [24]. Unfortunately, we are not able to advance the findings of those studies to reveal whether the source of these D-type symbionts was endogenous or environmental. *Symbiodinium* D are pandemic [21] and composed of several distinct groups with species-level genetic differentiation [33]. The D-type is rarely the dominant symbiont in corals, except in environments with high or variable temperatures and high sedimentation and/or turbidity [28,49]. On the GBR these environments are common on inshore reefs, such as Magnetic Island, and also occur in lagoons or other shallow, ponding reef systems [46]. It is increasingly recognized that cost–benefit relationships between coral and *Symbiodinium* associations are not only dependent on the symbiont type [8,33,50] but also the environment. For example, the cost in growth of corals that host D may be lost in warmer environments [51,52]. As human impacts on coral reefs intensify, the environmental conditions that promote the distribution and abundance of D may become more frequent and widespread [33]. Predicting the impacts of shifts towards D-dominated symbiont associations on coral health and resilience will require sensitive molecular tools to distinguish between very low abundance or novel uptake (including environmental abundance of these symbionts), as well as knowledge of host and symbiont genotype by environment interactions that may control the *in hospite* abundance of these types.

Our data highlight the importance of shuffling as a mechanism for corals that are not already dominated by tolerant D-type symbionts to recover from extreme summer heat stress and bleaching. Shuffling can shift stress thresholds within a short time period that is relevant to the rates of environmental change currently being experienced [5,22–24,48]. It is ecologically significant that 25% of colonies from the Keppels survived temperatures that far exceeded their historical temperature regimes (figure 1), bleaching and mortality thresholds [30,31]. All but one surviving colony either started off with D or became D-dominant during the course of the experiment (figure 5). The fact that half of the survivors shuffled supports this process as a mechanism of rapid acclimatization to rising temperatures [23,24]. Our results suggest that the background abundance was important and we estimated a minimum D : C threshold of 0.003 above which the relationship with S : H was more gradual than below (i.e. bleaching was less severe). Eighteen colonies from the Keppels population increased their D : C ratio over the course of the extreme heat exposure but failed to recover and died before the last sampling point; however, those that started above the threshold tended to live for longer and have higher final S : H ratios. Two Davies Reef colonies had pre-summer D : C ratios above the 0.003 threshold level but did not shuffle nor survive (figure 5). Clearly, there is still much to learn about the role of background symbionts in the physiological adaptation potential of coral populations under warmer and more variable conditions.

It is well established that the identity and diversity of *Symbiodinium* complements greatly affect fundamental aspects of the ecology of their coral hosts including growth rate, resistance to and recovery from environmental stress [3,33] and that multiple *Symbiodinium* types may be present in low abundance in many (or possibly the majority) of coral species (reviewed in [3]). Sensitivity modelling by Fabini [53] found higher stability (defined as the duration and number of species that persisted through environmental perturbations) of coral communities with more diverse symbiont complements probably because they provide redundant or complementary functions and increased the potential for local recovery following disturbance. It follows that an objective for natural resource management would be to identify and conserve species, locations or environmental conditions where symbiont diversity is high. Data on both high- and low-abundance *Symbiodinium* types can, therefore, provide valuable information on the status and trend of the health and resilience of corals and could provide baseline information for integrated coral reef monitoring and adaptive management [54]. The diversity and abundance of symbiont complements can be routinely and inexpensively screened with next-generation sequencing methods [18] and could feasibly be analysed on large spatial, temporal and taxonomic scales. Moreover, cataloguing of symbiont diversity and abundance within databases and visualization tools such as SymBioGBR and eAtlas provide perspectives that are relevant for natural resource management [55,56]. This approach would build integrated knowledge of the status and trend in coral symbiosis and health and (in time) provide a resource to evaluate the cost and benefits of management actions under different environmental scenarios [57,58].

## Supplementary Material

Supplementary Material for “Recovery from bleaching is mediated by threshold densities of background thermo-tolerant symbiont types in a reef-building coral.” 1. Laboratory procedures a. Symbiont genotyping and abundance estimation b. Copy number estimation 2. Statistical procedures a. Calculation of relative symbiont abundance b. Data transformation and binning procedure c. Model fitting 3. Additional supporting results 4. References

## Data accessibility

The associated data of this study are accessible on the Dryad Digital Repository (http://dx.doi.org/10.5061/dryad.t6p8c).
