# Supplementary material for: Recovery from bleaching is mediated by threshold densities of background thermo-tolerant symbiont types in a reef-building coral
Source: R Soc Open Sci. 2016 Jun 29;3(6):160322. doi: 10.1098/rsos.160322 (PMC4929921; doi:10.1098/rsos.160322)
Supplement: Supplementary Material for “Recovery from bleaching is mediated by threshold densities of background thermo-tolerant symbiont types in a reef-building coral.” 1. Laboratory procedures a. Symbiont genotyping and abundance estimation b. Copy number estimation 2. Statistical procedures a. Calculation o [file rsos160322supp1.docx]

Supplementary Material for “Recovery from bleaching is mediated by threshold densities of background thermo-tolerant symbiont types in a reef-building coral*.”*

1. Laboratory procedures
   1. Symbiont genotyping and abundance estimation
   2. Copy number estimation
2. Statistical procedures
   1. Calculation of relative symbiont abundance
   2. Data transformation and binning procedure
   3. Model fitting
3. Additional supporting results
4. References

1. Laboratory procedures:

(a) Symbiont genotyping and abundance estimation

DNA was extracted from ground ultra frozen 100% EtOH preserved samples. Extraction of DNA was performed using a MoBio plant kit with modifications to the manufacturer’s protocol. Specifically, extraction lysate was homogenised with two minutes of beating in the presence of 3 1 - 2mm stainless steel beads. The homogenate was the centrifuged for one minute at 13,000 rpm to precipitate skeletal material and the supernatant was used in subsequent extractions.

The dominant symbiont type was determined in 330 samples with DGGE of the ITS-2 region ([1](#_ENREF_1)) and the relative abundance C and D types was quantified with quantitative PCR (qPCR) with specific primers for the Actin locus ([2](#_ENREF_2)). For the DDGE genotyping, we amplified the *Symbiodinium* ITS2 region using primers and a touchdown thermal cycle described in LaJeunesse ([1](#_ENREF_1)). PCR products and known reference samples were applied to a denaturing gel on an Ingeny electrophoresis system (Ingeny, The Netherlands) and run for 20 hrs at 75 volts using a denaturing gradient of 30 – 50%. Gels were stained with SYBR-green (Life Technologies, VIC, Australia) prior to imaging and gel band excision. Gel bands for sequencing were punched out with a cut pipette tip and placed directly into a pre-prepared tube containing PCR master mix with the same DGGE primers minus GC clamp and were re-amplified using the thermal cycle protocol as above. Sequencing was performed by Macrogen (Korea) and sequence analysis undertaken with MEGA5 ([3](#_ENREF_3)) after manual removal of low and bad signal. Nine samples representing the diversity of DGGE banding patterns across all samples were selected and three bands from each sample were sequenced. ITS2 sequence analysis distinguished C1, C3 and C131 but could not differentiate between D, D1 or D1a types ([1](#_ENREF_1)) and hereafter referred to as “D”. Co-occurring C and D symbiont types were detected in 21 samples and two C types (C1 and C131) was distinguished in one C1 dominant sample (D1 in Jan 2006). This sample was CNR corrected with C1 copy number.

Quantitative PCR master mixes for amplification of the *Actin* locus in the coral host, *Symbiodinium* clade C and *Symbiodinium* clade D were prepared with Sensi-Mix SYBR No-Rox mastermix (Bioline, NSW, Australia) with primers described in Mieog et al. ([2](#_ENREF_2)) at 500 nmol per reaction. A liquid handling robot (CAS1200, Qiagen, VIC, Australia) was utilized to prepare each qPCR run by adding 15µl of master mix and 5 µl of DNA template prepared at between 1 and 10 ng / µl giving a total reaction volume of 20 µl. Each combination of master mix and DNA template was assayed in triplicate with appropriate negative controls. The thermal cycling protocol consisted of 10 min at 95°C then 40 cycles with two steps of 95°C for 10 sec followed by 60°C for 30 sec. For all runs, C_T_ values were measured with the fluorescence threshold set at 0.1. Efficiency curves were conducted with samples that contained only D or only C. Briefly, two samples containing only clade D (K1, 30-Apr-2005) and clade C3 (K24, 29-Mar-2005) as determined via DGGE, quantitative PCR and sequence comparison were mixed in equal amounts and serially diluted. Reaction efficiencies for symbionts and the coral host locus ranged from 0.97 to 1.03.

(b) Copy number estimation

For determination of specific symbiont or symbiont/host ratios it is necessary to correct for copy number for the *Actin* region of interest in each of the *Symbiodinium* clades or types present. Actin copy number was determined in coral samples previously genotyped to host ITS2 symbiont types C1, C3 and C131 (accession numbers KJ612067 – 069 respectively). Firstly, the *Actin* locus was amplified in samples with PCR using the primers described above. PCR conditions included a denaturation step at 95°C for 10 minutes, 35 cycles (touchdown mode) of 95°C for 40 seconds, 60°C for 40 seconds and 72°C for 30 seconds with the annealing temperature decreasing by 0.5°C every cycle for the first 20 cycles and maintained at 50°C for the remaining 15 cycles with a final extension at 72°C for 10 minutes. Products from PCR’s were cloned into TOPO vectors according to the manufacturer’s instructions (Life Technologies, Victoria, Australia). Selected clones were analysed for the *Actin* insert and positive clones used to generate plasmid DNA for standard curves ranging from 10^6^ to 10^1^ copies. *Symbiodinium* was isolated from *Acropora millepora* (Keppels, n = 3), *Acropora millepora* (Davies reef, n = 3) and *Acropora tenuis* (Magnetic Island, n = 3) known to harbour clade C3, C131 and C1 respectively. DNA was extracted from a known cell number (10^5^ - 10^6^ cells) and a 10-fold dilution series prepared from these extracts. Comparison of the Ct values between known cell numbers and known copies allowed for the *Actin* copy number to be calculated. The results were: clade C3 = 1.0 ± 0.5, C1= 5.1 ± 1.2 and C131 = 4.3 ± 1.0 (mean ± SD). The copy number for C1 was within the margin of error of that determined by Mieog et al. ([2](#_ENREF_2)) for the same symbiont type derived from the same coral species at Magnetic Island which was 7.0 ± 2.9. For clade D, we used the copy number of 1.0 ± 0.7 as determined by Mieog et al. ([2](#_ENREF_2)).

2. Statistical procedures

All data and scripts have been deposited in Dryad ([4](#_ENREF_4))

(a) Symbiont abundance estimation

Symbiont types C or D to host ratios were calculated with the formula S_[C or D]_:H ratio = [2^C^_T_^(H)-C^_T_^(S^_[C or D]_^)^ /actin copies per cell] * 2, where C_T_(H) and C_T_(S_[C or D]_) are the C_T_ values of the coral host and symbiont-specific reactions. For clade C and D, the *Actin* copy number per cell is as described above with a final multiplier of two due to the presence of haploid *Symbiodinium* cells ([5](#_ENREF_5)) and diploid *A. millepora* cells. The overall symbiont:host (S:H) ratio was obtained from the sum of clade C and D ratios (S_[C]_:H + S_[D]_:H). The extraction efficiency between coral host and *Symbiodinium* was similar when a non-SDS extraction method was used ([6](#_ENREF_6)). We used a guanidine thiocyanate based spin column extraction method and hence deemed it appropriate to assume a similar extraction efficiency of coral host and symbiont DNA.

Ratios of *Symbiodinium* clade D:C were calculated with the formula D:C ratio = [2^C^_T_^(clade C)-C^_T_^(clade D)^ * CNR], where C_T_ (clade C) and C_T_ (clade D) are the C_T_ values of clade C and clade D symbionts respectively and CNR is the copy number ratio of clade C to clade D. Since samples were identified by DGGE to be dominated by different C-types, the CNR for individual samples was based on their specific C type.

Data transformation

The S:H ratios were skewed because of the formula used for their calculation, and as a consequence so was our D:C ratio metric. Standard square root and logarithmic transformations failed to normalise the data and reduced the variance in the Davies and Magnetic Island populations (Supporting Material Figure 1). As a consequence we could not obtain good model fits for these populations. We therefore applied a custom transformation that consisted of 20 unequally sized bins (Supplementary Table 1). This approach normalised the data and had the advantage that it provided adequate granularity of the data particularly for low D:C estimates of interest here. It also naturally constrained the data between 0 and 1 (i.e., all C or all D) when samples with dominant D but background C would otherwise produce ratios with an upper bound of infinity.

Supplementary Figure 1: Square root transformed ratio of D to C symbionts across an annual cycle that included a very warm summer and coral bleaching.

Supplementary Table 1: Bin categories for symbiont D and C ratios and the number of samples of each population in each bin for all time points combined.

| Bin # | Minimum D:C ratio | Number of samples | | |
| --- | --- | --- | --- | --- |
|  |  | Keppels | Davies Rf | Magnetic Is |
| 1 | 0 (Pure C) | 40 | 48 | 0 |
| 2 | 0.0001 | 1 | 57 | 0 |
| 3 | 0.0005 | 2 | 23 | 0 |
| 4 | 0.001 | 3 | 1 | 0 |
| 5 | 0.003 | 7 | 0 | 0 |
| 6 | 0.005 | 3 | 3 | 0 |
| 7 | 0.01 | 2 | 0 | 0 |
| 8 | 0.05 | 2 | 2 | 0 |
| 9 | 0.1 | 3 | 0 | 0 |
| 10 | 0.3 | 0 | 1 | 0 |
| 11 | 0.5 | 3 | 0 | 0 |
| 12 | 0.7 | 2 | 0 | 0 |
| 13 | 0.9 | 0 | 2 | 0 |
| 14 | 1 (50:50) | 1 | 0 | 0 |
| 15 | 2 | 2 | 1 | 0 |
| 16 | 10 | 4 | 0 | 0 |
| 17 | 100 | 4 | 0 | 0 |
| 18 | 1000 | 3 | 0 | 4 |
| 19 | 10000 | 5 | 0 | 4 |
| 20 | ∞(Pure D) | 40 | 0 | 93 |

b. Model fitting

Generalized change-point (piece-wise) ([7](#_ENREF_7)) regression models were used to model December S:H ratio (i.e., bleaching level) against April and September D:C ratios according to:

$$y_{i}\sim QuasiPoisson\left( \eta_{i} \right)$$

$$\log\left( \eta_{i} \right)= \beta_{0}+\beta_{1}I(x_{i}>x_{cp})(x_{i})+ \beta_{2}I(x_{i}>x_{cp})(x_{i}-x_{cp})$$

where $\log\left( \eta_{i} \right)$ represents a log-link function relating the model residuals to a Quasi-Poisson distribution, *y_i_* and *x_i_* are the observed values of the S:H ratio and D:C ratio respectively, and *x_cp_* is the value of the change-point.

The quasi-poisson distribution ([8](#_ENREF_8)) was considered appropriate as it naturally imposes a lower limit of 0 and copes with reasonable levels of anticipated over-dispersion. In the above model *I*(.) is an indicator function that evaluates to either 1 (i.e. xi < xcp is true) or 0 otherwise. β0, beta1 and β2 respectively represent (on a log-scale) the expected value of y when x is equal to the value of the change-point (xcp), and the rates of change in y per unit change in x relative to the change-point.

1. Additional Supporting Results:

We did not find a relationship between pre-summer symbiont density and bleaching level (i.e., the partial slopes for the relationship between S:H (C + D) in December and S:H (C + D) in April 2005 and population interactions were Keppels: b=-0.08, t_62_=-1.437, P>0.05; Magnetic Island: b=0.139, t_62_=0.561, P>0.05; Davies Reef: b=-0.011, t_62_=-0.049, P>0.05). This result was similar with pre-summer estimates from September 2005 (Supplementary Figure 2).

Supplementary Figure 2: Pre-summer S:H ratio (i.e., April or September 2005) did not predict bleaching level in either of the three source populations (x , y = 0 , 0)

To identify the predictors of shuffling we examined the relationship between pre-experimental D:C ratios and mid-summer bleaching level (i.e., Sep 2005 D:C ratio vs Dec 2005 S:H ratio).Our analysis of A similar breakpoint was evident for the Keppel Island populations with the these data compared to the April 2005 data. We did not find a relationship between the D:C (September 2005) and S:H (December) for the Davies Reef or the Magnetic Island populations (Supplementary Figure 3).

Supplementary Figure 3: Level of bleaching expressed as symbiont abundance in summer as a function of pre-bleaching (September) D:C symbiont ratio.

4. References

1. LaJeunesse TC. Diversity and community structure of symbiotic dinoflagellates from Caribbean coral reefs. Marine Biology. 2002;141(2):387-400.

2. Mieog JC, Van Oppen MJH, Berkelmans R, Stam WT, Olsen JL. Quantification of algal endosymbionts (*Symbiodinium*) in coral tissues using real-time PCR. Molecular Ecology Resources. 2009;9(1):74-82.

3. Tamura K, Peterson D, Peterson N, Stecher G, Nei M, Kumar S. MEGA5: Molecular Evolutionary Genetics Analysis Using Maximum Likelihood, Evolutionary Distance, and Maximum Parsimony Methods. Mol Biol Evol. 2011;28(10):2731-9.

4. Data from: Recovery from bleaching is mediated by threshold densities of background thermo-tolerant symbiont types in a reef-building coral. [database on the Internet]. Dryad Digital Repository. dx.doi.org/10.5061/dryad.t6p8c). 2016.

5. Santos SR, Coffroth MA. Molecular genetic evidence that dinoflagellates belonging to the genus Symbiodinium freudenthal are haploid. Biol Bull. 2003;204(1):10-20.

6. Mieog JC, Van Oppen MJH, Berkelmans R, Stam WT, Olsen JL. Quantification of algal endosymbionts (Symbiodinium) in coral tissue using real-time PCR. Molecular Ecology Resources. 2009;9(1):74-82.

7. Toms JD, Lesperance ML. Piecewise regression: A tool for identifying ecological thresholds. Ecology. 2003;84(8):2034-41.

8. Wolfinger R, O’Connell M. Generalized linear mixed models a pseudo-likelihood approach. Journal of Statistical Computation and Simulation. 1993;48:233–43.
